# Evaluation of Menstrual Cycle Tracking Behaviors in the Ovulation and Menstruation Health Pilot Study: Cross-Sectional Study

**DOI:** 10.2196/42164

**Published:** 2023-10-27

**Authors:** Tatheer Adnan, Huichu Li, Komal Peer, Elizabeth Peebles, Kaitlyn James, Shruthi Mahalingaiah

**Affiliations:** 1 Harvard School of Public Health Boston, MA United States; 2 Massachusetts General Hospital Boston, MA United States

**Keywords:** reproductive informatics, menstrual health tracking apps, digital epidemiology, women's health, mobile health, mHealth, gynecology, health application, tracking health app, menstrual health, reproductive health

## Abstract

**Background:**

Menstrual cycle tracking apps (MCTAs) have potential in epidemiological studies of women’s health, facilitating real-time tracking of bleeding days and menstrual-associated signs and symptoms. However, information regarding the characteristics of MCTA users versus cycle nontrackers is limited, which may inform generalizability.

**Objective:**

We compared characteristics among individuals using MCTAs (app users), individuals who do not track their cycles (nontrackers), and those who used other forms of menstrual tracking (other trackers).

**Methods:**

The Ovulation and Menstruation Health Pilot Study tested the feasibility of a digitally enabled evaluation of menstrual health. Recruitment occurred between September 2017 and March 2018. Menstrual cycle tracking behavior, demographic, and general and reproductive health history data were collected from eligible individuals (females aged 18-45 years, comfortable communicating in English). Menstrual cycle tracking behavior was categorized in 3 ways: menstrual cycle tracking via app usage, that via other methods, and nontracking. Demographic factors, health conditions, and menstrual cycle characteristics were compared across the menstrual tracking method (app users vs nontrackers, app users vs other trackers, and other trackers vs nontrackers) were assessed using chi-square or Fisher exact tests.

**Results:**

In total, 263 participants met the eligibility criteria and completed the digital survey. Most of the cohort (n=191, 72.6%) was 18-29 years old, predominantly White (n=170, 64.6%), had attained 4 years of college education or higher (n= 209, 79.5%), and had a household income below US $50,000 (n=123, 46.8%). Among all participants, 103 (39%) were MCTA users (app users), 97 (37%) did not engage in any tracking (nontrackers), and 63 (24%) used other forms of tracking (other trackers). Across all groups, no meaningful differences existed in race and ethnicity, household income, and education level. The proportion of ever-use of hormonal contraceptives was lower (n=74, 71.8% vs n=87, 90%, *P*=.001), lifetime smoking status was lower (n=6, 6% vs n=15, 17%, *P*=.04), and diagnosis rate of gastrointestinal reflux disease (GERD) was higher (n=25, 24.3% vs n=12, 12.4%, *P*=.04) in app users than in nontrackers. The proportions of hormonal contraceptives ever used and lifetime smoking status were both lower (n=74, 71.8% vs n=56, 88.9%, *P=*.01; n=6, 6% vs n=11, 17.5%, *P=*.02) in app users than in other trackers. Other trackers had lower proportions of ever-use of hormonal contraceptives (n=130, 78.3% vs n=87, 89.7%, *P*=.02) and higher diagnostic rates of heartburn or GERD (n=39, 23.5% vs n=12, 12.4%, *P*.03) and anxiety or panic disorder (n=64, 38.6% vs n=25, 25.8%, *P=*.04) than nontrackers. Menstrual cycle characteristics did not differ across all groups.

**Conclusions:**

Our results suggest that app users, other trackers, and nontrackers are largely comparable in demographic and menstrual cycle characteristics. Future studies should determine reasons for tracking and tracking-related behaviors to further understand whether individuals who use MCTAs are comparable to nontrackers.

## Introduction

By allowing users to track their bleeding days and associated signs and symptoms in real time, menstrual cycle tracking apps (MCTAs) facilitate prospective data collection on menstrual cycle characteristics in the general population [1]. Recently, the application of such data in epidemiological research has become a topic of interest. Existing research has used large app-based menstrual cycle data to examine and characterize the menstrual cycle in the population, examine associations between menstrual cycle characteristics (eg, cycle length and variability) and symptoms, and explore the potential of such self-tracking data in phenotyping endometriosis and predicting polycystic ovary syndrome (PCOS) among patient populations [2-4]. The relatively large sample size, diverse user population, and accessibility have made MCTA data potentially desirable for population-based research on women’s health [5]. However, data collected from MCTAs also have notable limitations and challenges such as missing data, loss to follow-up, and the questionable generalizability of MCTA users to the free-living menstruating population [5,6]. So far, studies using the app-based cycle tracking data have provided limited information on their participants’ characteristics. To date, comparisons of demographic factors, health behaviors and conditions, and menstrual cycle characteristics of MCTA users and those who track their cycles with other methods or those who do not track their cycles is lacking [5].

The primary objective of this analysis was to compare demographic factors, health behaviors and conditions, and certain menstrual cycle characteristics between individuals tracking their cycles through MCTAs (app users) and those who do not track their cycles (nontrackers; comparison 1). We further explored differences in the same variables in 2 secondary analyses between app users and those who use other forms of menstrual cycle tracking (any trackers; comparison 2), and between any trackers and nontrackers (comparison 3).

## Methods

### Study Design and Data Collection

The data used in this study were deidentified and retrieved from the parent Ovulation and Menstruation (OM) Health Pilot Study. The OM Health Pilot Study was conducted primarily to determine the feasibility of enrolling participants from diverse backgrounds using varied recruitment modalities. This study used survey questionnaires to collect information about demographic factors, health conditions and behaviors, and menstrual cycle characteristics at different life stages [7]. The study originally recruited female participants aged 18-45 years between September 2017 and March 2018 from 3 sources: in-clinic recruitment, recruitment at community events, and internet-based recruitment. In-clinic recruitment occurred at the Boston Medical Center (Boston, Massachusetts), and community recruitment was conducted at the Boston Women’s Market (Boston) on September 17, 2017. Internet-based recruitment methods included sending out email communications, creating study social media engagement accounts, and sharing recruitment material in individual social networks of the study staff. The study website and social media pages were discoverable on any internet search. Women who were pregnant, had undergone hysterectomy or oophorectomy, experienced amenorrhea due to chemotherapy, or were unwilling or unable to provide an email address at the time of the survey were deemed ineligible for participation. Eligible participants completed a web-based survey with questions to ascertain their demographic factors, health behaviors and conditions, and menstrual cycle characteristics. Detailed descriptions of the study design, recruitment, enrollment, and survey completion can be found in our previously published work [7].

**Figure 1:**
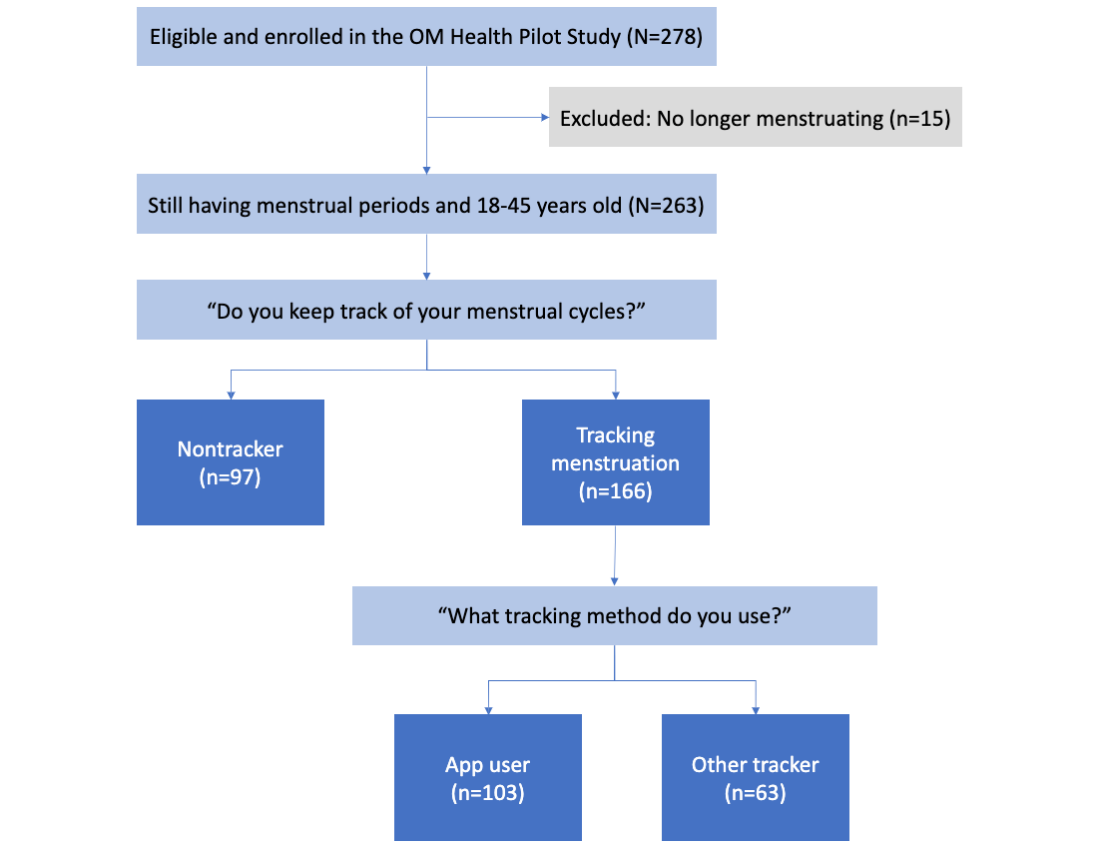
Flowchart of participant recruitment in the study. OM: Ovulation and Menstruation.

Demographic factors were surveyed to determine race and ethnicity, education status, income category, and anthropometric measures such as height and weight. Health behaviors were assessed, including alcohol consumption and tobacco smoking habits. Self-reported clinician diagnosed health conditions were surveyed, including questions for chronic diseases (hypertension, diabetes, gastroesophageal reflux disease [GERD], eating disorder, high cholesterol, nonalcoholic fatty liver disease, thyroid disease, excess prolactin, sleep apnea, posttraumatic stress disorder [PTSD], chronic fatigue syndrome, seizure disorder, depression, and anxiety or panic disorder), reproductive health (history of pregnancy, PCOS status, uterine fibroids, endometriosis, and premature ovarian failure), and hormone use (eg, current hormone use, use of hormones in the past including the timing of the start and end of hormone use, types of hormone use, and reasons for hormone use). Health behaviors were assessed, including alcohol consumption and tobacco smoking habits. Menstrual cycle characteristics including time to cycle regularity, age at menarche, cycle length, cycle regularity, and categorized bleed day duration were ascertained. Menstrual cycle tracking behavior and methods of cycle tracking, including MCTAs, paper (or written) calendars, digital calendars, computer software, hormonal birth control use, memory, or other, were obtained. The analytic cohort in this study consisted of participants who had answered questions on menstrual cycle tracking (Figure 1). The survey is available on the Harvard Dataverse [8].

### Menstrual Tracking Behavior

Participants were categorized into 3 groups: participants who use mobile apps to track their menstrual cycles (app users), those who do not track their menstrual cycles (nontrackers), and those who track their menstrual cycles using other methods (other trackers). The primary analysis focused on the differences in demographic factors, health behaviors and conditions, and menstrual cycle characteristics between app users and nontrackers (comparison 1). Additional comparisons between app users and other trackers (comparison 2) and between all trackers (app users and other trackers) and nontrackers are shown in Multimedia Appendix 1 (comparison 3).

### Participant Characteristics

Key metrics compared among menstrual tracking categories included age (categorized as 18-29, 30-39, and 40-45 years), BMI (underweight [<18.5 kg/m^2^], normal weight [18.5-24.9 kg/m^2^], overweight [25.0-29.9 kg/m^2^], and obese [≥30.0 kg/m^2^]), race (White, Hispanic, Black, Asian, and more than one race), household income (below US $25,000, US $25,000 to $49,999, US $50,000 to $74,999, US $75,000 to $99,999, US $100,000 or more, prefer not to answer, and don’t know), education attainment (high school graduate or General Educational Development or lesser education, some college or 2-year degree, 4-year college graduate, and more than a 4-year college degree), the prevalence of health behaviors and conditions, and menstrual cycle characteristics (age at menarche, time to cycle regularity, cycle length, cycle regularity, and categorized bleed day duration). Time to cycle regularity was categorized as less than 1 year, 1-2 years, 3-4 years, 5 years or more, or never. Age at menarche was categorized as integers. Menstrual cycles were categorized as less than 24 days, between 24 and 38 days, and longer than 38 days. Bleeding duration was categorized as 1-3 days or 4-7 days; no participants reported a bleeding duration over 7 days.

### Statistical Analysis

Differences in demographic factors, health behaviors and conditions, and menstrual cycle characteristics in comparison 1 (primary analysis) were assessed using chi-square tests (or the Fisher exact test when appropriate due to small cells); a *P* value of less than .05 was considered significant. The same analyses were performed for comparisons 2 and 3 (secondary analyses).

### Ethical Considerations

This research was deemed nonhuman subjects research by the institutional review board (IRB20-0623) at Harvard T.H. Chan School of Public Health.

## Results

### Demographic Factors

Of the 263 women who were eligible and completed the required questions for these analyses, 36 (14%) had been recruited from the clinic, 61 (23%) from the community fair, and 166 (63%) from the internet. Among them, 103 (39%) were app users, 97 (37%) were nontrackers, and 63 (24%) were other trackers. While the majority of individuals were recruited from the internet (n=166, 63%), the trend in tracking status by recruitment location was not significant (test for trend *P=*.08; Table S1 in Multimedia Appendix 1). The majority of the cohort (n=191, 72.6%) was 18-29 years old, predominantly White (n=170, 64.6%), had attained 4 years of college education or higher (n=209, 79.5%), and had a household income below US $50,000 (n=123, 46.8%). Of note, similar distributions of these demographic factors were observed across menstrual tracking categories, as summarized in Table 1.

**Table 1:** Demographic factors by menstrual tracking categories.

Characteristics		Participants, n (%)			
		Included cohort (N=263)	App users (n=103)	Nontrackers (n=97)	Other trackers (n=63)
**Age category (years)**					
	18-29	191 (72.6)	76 (73.8)	73 (75.3)	42 (66.7)
	30-39	59 (22.4)	22 (21.4)	22 (22.7)	15 (23.8)
	40-45	13 (4.9)	5 (4.9)	2 (2.1)	6 (9.5)
**Race**					
	White	170 (64.6)	67 (65.0)	63 (64.9)	40 (63.5)
	Hispanic	17 (6.5)	5 (4.9)	8 (8.2)	4 (6.3)
	Black	31 (11.8)	15 (14.6)	11 (11.3)	5 (7.9)
	Asian	19 (7.2)	7 (6.8)	5 (5.2)	7 (11.1)
	More than one race	24 (9.1)	9 (8.7)	8 (8.2)	7 (11.1)
	Prefer not to answer	2 (0.8)	0 (0)	2 (2.1)	0 (0)
**Education**					
	High school graduate or General Educational Development	16 (6.1)	5 (4.9)	7 (7.2)	4 (6.3)
	Some college or 2-year degree	35 (13.3)	14 (13.6)	15 (15.5)	6 (9.5)
	4-year college graduate	96 (36.5)	42 (40.8)	32 (33.0)	22 (34.9)
	More than a 4-year college degree	113 (43.0)	42 (40.8)	40 (41.2)	31 (49.2)
	Unknown	3 (1.1)	0 (0)	3 (3.1)	0 (0)
**Annual household income (US $)**					
	<25,000	56 (21.3)	19 (18.4)	20 (20.6)	17 (27.0)
	25,000-49,999	67 (25.5)	26 (25.2)	27 (27.8)	14 (22.2)
	50,000-74,000	43 (16.3)	17 (16.5)	16 (16.5)	10 (15.9)
	75,000-99,999	19 (7.2)	10 (9.7)	7 (7.2)	2 (3.2)
	≥100,000	41 (15.6)	15 (14.6)	13 (13.4)	13 (20.6)
	Prefer not to answer	16 (6.1)	7 (6.8)	5 (5.2)	4 (6.3)
	Don’t know	21 (8.0)	9 (8.7)	9 (9.3)	3 (4.8)

### Health Behaviors and Conditions

#### Overview

Among all participants, a total of 32 (12%) women reported having smoked >100 cigarettes. Regarding reproductive health history, 16% (n=42) of participants reported having been pregnant before, 14% (n=37) of them reported doctor-diagnosed PCOS, and 8% (n=22) of them reported self-diagnosed PCOS. The prevalence of uterine fibroids and endometriosis was 6% (n=16) and 5% (n=9), respectively, in our study population. The use of hormonal contraceptives was notable with 83% (n=217) of all participants reporting current or past use. Regarding other medical health histories, we found that 34% (n=89) and 31% (n=81) of all participants reported anxiety or panic disorder and depression, respectively. Other conditions that had more than 10% prevalence in the study population included GERD (n=51, 19%), hypercholesterolemia (n=28, 11%), and PTSD (n=28, 11%).

#### Comparison 1: App Users Versus Nontrackers

When comparing health behaviors and conditions between app users and nontrackers, we found several notable differences. A greater percentage of nontrackers (n=87, 90%) reported ever-use of hormonal contraceptives than app users (n=74, 71.8%; Table 2). Smoking habits also differed significantly between the 2 groups (*P=*.04)—while only 6% (n=6) of app users had smoked >100 cigarettes over their lifetime, 17% (n=15) of nontrackers had smoked >100 cigarettes. The prevalence of certain health conditions also differed between app users and nontrackers—heartburn (or GERD) diagnosis occurred at a higher rate among app users (n=25, 24.3%) than among nontrackers (n=12, 12.4%; *P=*.04). The prevalence of other health behaviors and conditions, including reproductive health conditions, did not differ significantly between the 2 groups (Table 2).

**Table 2:** Comparison 1: health behaviors and conditions between app users (n=103) and nontrackers (n=97).

		App users, n (%)	Nontrackers, n (%)	*P* value^a^	
Smoked at least 100 cigarettes over their lifetime		6 (6)	15 (17)	.04	
**BMI categories**					.63
	Underweight (<18.5 kg/m^2^)	3 (2.9)	3 (3.1)		
	Normal weight (18.5-24.9 kg/m^2^)	58 (56.3)	52 (53.6)		
	Overweight (25.0-29.9 kg/m^2^)	12 (11.7)	18 (18.6)		
	Obese (≥30.0 kg/m^2^)	29 (28.2)	22 (22.7)		
	Unknown	1 (1.0)	2 (2.1)		
PCOS^b^ diagnosis by a doctor		17 (17)	10 (11)	.22	
PCOS self-diagnosis		10 (9.7)	7 (8)	.45	
Ever pregnant		16 (15.5)	14 (14.4)	.84	
Ever-use of hormonal contraceptives		74 (71.8)	87 (90)	.001	
**Participants’ rating of current health**					.82
	Excellent, very good, or good	91 (88.3)	86 (88.7)		
	Fair or poor	11 (10.7)	9 (9.3)		
	Unknown	1 (1.0)	2 (2.1)		
Uterine fibroids diagnosis		6 (6)	5 (5)	>.99	
Endometriosis diagnosis		1 (1)	5 (5)	.11	
Premature ovarian failure diagnosis		0 (0)	0 (0)	N/A^c^	
Heartburn or GERD^d^ diagnosis		25 (24.3)	12 (12.4)	.04	
Eating disorder diagnosis		13 (13)	9 (10)	.51	
Hypertension diagnosis		5 (4.9)	2 (2.1)	.45	
Hypercholesterolemia diagnosis		9 (8.7)	6 (6.2)	.59	
Diabetes diagnosis		2 (1.9)	0 (0)	.50	
Nonalcoholic fatty liver disease diagnosis		1 (1.0)	0 (0)	.85	
Thyroid disease diagnosis		4 (3.9)	3 (3.1)	.63	
Excess prolactin diagnosis		1 (1)	0 (0)	>.99	
Sleep apnea diagnosis		5 (5)	1 (1)	.21	
PTSD^e^ diagnosis		10 (9.4)	8 (8.2)	.81	
Chronic fatigue syndrome diagnosis		1 (1)	0 (0)	>.99	
Seizure disorder diagnosis		2 (2)	0 (0)	.50	
Depression diagnosis		31 (30.1)	26 (29)	.64	
Anxiety or panic disorder diagnosis		36 (35.0)	25 (25.8)	.16	

^a^*P* values were calculated using the Fisher exact test due to small cells when prevalence was below 10%.

^b^PCOS: polycystic ovary syndrome.

^c^N/A: not applicable.

^d^GERD: gastrointestinal reflux disease.

^e^PTSD: posttraumatic stress disorder.

#### Comparison 2: App Users Versus Other Trackers

When comparing app users to other trackers, similar findings with smoking habits and ever-use of hormonal contraceptives were observed. Other trackers had significantly higher reports of smoking at least 100 cigarettes over their lifetime (*P=*.02) and higher reports of hormonal contraceptive ever-use (*P=*.01) than app users (Table S2). In addition, a diagnosis of hypercholesterolemia was more prevalent among other trackers (n=13, 20.6%) than among app users (n=9, 8.7%; *P=*.04). No differences in the prevalence of other health behaviors or conditions were noted (Table S2 in Multimedia Appendix 1).

#### Comparison 3: Other Trackers Versus Nontrackers

When comparing other trackers to nontrackers, other trackers were less likely to report ever-use of hormonal contraceptives (n=130, 78.3% vs n=87, 89.7%, *P=*.02), more likely to have a heartburn or GERD diagnosis (n=39, 23.5% vs n=12, 12.4%, *P=*.03), and more likely to have an anxiety or panic disorder diagnosis (n=64, 38.6% vs n=25, 25.8%, *P=*.04; Table S3 in Multimedia Appendix 1).

### Menstrual Cycle Characteristics

#### Overview

The overall cohort’s age at menarche and time to cycle regularity were 12 years (n=73, 27.8%) and <1 year (n=134, 51%), respectively (Table S4). The majority of the cohort had regular periods occurring every 24-38 days (n=181, 68.8%), had experienced some irregularity at some point (n=158, 60.1%), and had a menstrual cycle bleeding duration of 4-7 days (n=144, 54.8%; Table S4 in Multimedia Appendix 1).

#### Comparison 1: App Users Versus Nontrackers

No differences were noted in the studied menstrual cycle characteristics between app users and nontrackers (Table 3). The most common age at which both nontrackers and app users experienced their first menstrual cycle was 12 years (n=24, 24.7%), and more than 60% of participants in both groups experienced regular menstrual cycles—ie, the menstrual cycle regularizing in <2 years after the onset of menarche (app users: n=76, 73.8%; nontrackers: n=65, 67%; Table S4 in Multimedia Appendix 1). Although most participants in both groups (app users: n=74, 71.8%; nontrackers: n=60, 61.9%) reported a normal cycle frequency of 24-38 days, we found that app users had a slightly higher prevalence of experiencing a cycle length of >38 days (n=11, 10.7%) than nontrackers (n=4, 4.1%), though this was not significant (*P*=.40).

**Table 3:** Comparison 1: cycle regularity, age at menarche, and cycle frequency between app users (n=103) and nontrackers (n=97).

		App users, n (%)	Nontrackers, n (%)	*P* value^a^	
**Age at menarche (years)**					.55
	≤7	1 (1.0)	0 (0)		
	8-10	12 (11.7)	8 (8.2)		
	11	19 (18.4)	22 (22.7)		
	12	31 (30.1)	24 (24.7)		
	13	24 (23.3)	17 (17.5)		
	14	7 (6.8)	11 (11.3)		
	15	6 (5.8)	6 (6.2)		
	≥16	2 (1.9)	5 (5.2)		
	Missing	1 (1.0)	4 (4.1)		
**Time to cycle regularity**					.67
	<1 year	59 (57.3)	45 (46.4)		
	1-2 years	17 (16.5)	20 (20.6)		
	3-4 years	5 (4.9)	5 (5.2)		
	≥5 years	6 (5.8)	5 (5.2)		
	Never	15 (14.6)	19 (19.6)		
	Missing	1 (1.0)	3 (3.1)		
**Period regularity**					.40
	<24 days	5 (4.9)	4 (4.1)		
	24-38 days	74 (71.8)	60 (61.9)		
	≥38	11 (10.7)	4 (4.1)		
	Missing	13 (12.6)	29 (29.9)		
**Period ever irregular**					.14
	No	44 (42.7)	31 (32)		
	Yes	59 (57.3)	64 (66)		
	Missing	0 (0)	2 (2.1)		
**Categorized bleed day duration**					.06
	0-3 days	40 (38.8)	50 (51.5)		
	4-7 days	62 (60.2)	45 (46.4)		
	Missing	1 (1)	2 (2.1)		

^a^*P* values were calculated using the Fisher exact test due to small cells when prevalence was below 10%.

#### Comparison 2: App Users Versus Other Trackers

No differences were noted in menstrual cycle characteristics between app users and other trackers (Table S5 in Multimedia Appendix 1).

#### Comparison 3: Other Trackers Versus Nontrackers

No differences were noted in age at menarche, time to cycle regularity, cycle length, and cycle regularity between other trackers and nontrackers. Other trackers were more likely to report bleeding durations of 4-7 days than nontrackers (other trackers: n=99, 59.6%; nontrackers: n=45, 46.4%; *P*=.05) and were more likely to report shorter bleed durations (0-3 days; other trackers: n=66, 39.8%; nontrackers: n=50, 51.5%; *P*=.05; Table S6 in Multimedia Appendix 1).

### Hormonal Contraceptive Ever Use

#### Comparison 1: App Users Versus Nontrackers

As noted above, ever-use of hormonal contraceptive differed significantly between app users and nontrackers (*P*=.001; Table 2). A greater proportion of nontrackers were currently using hormonal contraceptives (n=63, 72% vs n=31, 42%, *P<*.001; Table 4). In total, 34% (n=25) of app users and 47% (n=41) of nontrackers who had ever used hormonal contraceptives reported that using contraceptives achieved more regular, predictable periods (*P=*.09; Table 4).

**Table 4:** Comparison 1: hormonal contraceptive use between app users (n=74) and nontrackers (n=87) among those who have ever used hormonal contraceptives.

	App users	Nontrackers	*P* value
Current use of hormonal contraceptives, n (%)	31 (42)	63 (72)	<.001
Use of hormonal contraceptives to regulate periods, n (%)	25 (34)	41 (47)	.09

#### Comparison 2: App Users Versus Other Trackers

When comparing app users to other trackers who reported ever-use of hormonal contraceptives, a greater proportion of other trackers reported both current use of hormonal contraceptives (n=37, 66% vs n=31, 42%; *P*=.006) and use of contraceptives to achieve more regular periods (n=30, 54% vs n=25, 34%; *P*=.02; Table S7 in Multimedia Appendix 1).

#### Comparison 3: Other Trackers Versus Nontrackers

While a greater proportion of nontrackers reported current use of hormonal contraceptives than other trackers (n=63, 72% vs n=68, 52%; *P*=.003), there was no difference in the use of contraceptives for regulating periods between other trackers and nontrackers (n=55, 42% vs n=41, 47%; *P*=.48; Table S8 in Multimedia Appendix 1).

## Discussion

### Background

We compared participant characteristics including demographic factors, health behaviors and conditions, and menstrual cycle characteristics among women in the 3 groups. We present a primary comparison of MCTA users’ and nontrackers’ behavior, and secondary comparisons between app users and other trackers and between other trackers and nontrackers.

### Principal Findings

In this study, we found that app users, other trackers, and nontrackers are largely comparable in demographic factors and menstrual cycle characteristics. The significant differences among comparison groups were small overall and were only noted in health behaviors and conditions.

The most notable difference was the current use of hormonal contraceptives in comparison 1 (app users: n=31, 42%; nontrackers: n=63, 72%), comparison 2 (app users: n=31, 42%; other trackers: n=37, 66%), and comparison 3 (other trackers: n=68, 52%; nontrackers: n=63, 72%). Given that hormonal contraceptives can alter the user’s menstrual pattern to enable predicting bleeding days, participants in the other tracker or nontracker cohorts may not need to use MCTAs to monitor their menstrual status. A previous study on reasons for menstrual tracking indicated that improvement in knowledge concerning menstrual cycles, preparing for upcoming periods, and tracking cycle regularity and symptoms are key motivators to use menstrual health tracking apps [9]. We grounded our explanations for differences in current hormonal contraceptive use based on motivations for MCTA use.

Furthermore, while not significant, comparison 3 revealed a trend toward a shorter menstrual bleeding duration among nontrackers than among other trackers. This is congruent with the potential influence of hormonal contraceptive use (other trackers: n=68, 52%; nontrackers: n=63, 72%) on menstrual bleed length. The duration of menstrual bleeding, which occurs during the placebo week among oral contraceptive pill users, is considered a withdrawal bleed and may be lighter or different from an unmedicated menstrual bleed due to suppressive effects on the endometrium. Notably, the use of oral contraceptive pills may suppress ovulation and alter endometrial characteristics such as endometrial thickness.

### Study Strengths and Implications

An increasing number of studies have examined menstrual cycle characteristics using survey-based, and more recently, MCTA-retrieved data [10]. While such studies are crucial to furthering our understanding of characteristics that constitute regular cycles and those that may be indicative of menstrual disorders, limited focus has been placed on understanding the fundamental differences between women using MCTAs from those who do not. Since researchers may not be able to ensure that no differences exist, qualifying the nature of differences that may exist between these groups is crucial in adjusting and accounting for potential selection biases that may occur.

The strengths of this study are that demographics, health behaviors and conditions, and menstrual cycle characteristics were ascertained across tracking behavior categories, thereby providing preliminary insights into similarities and differences between app users and nontrackers.

### Limitations

Our findings must be interpreted with the following limitations. The first limitation of our study was the small sample size of 263 participants. Second, we required an email address, which may limit enrollment as possession of a smartphone, internet access, and an email address might not be universal. This introduced a potential selection bias that limited the generalizability of the recruited sample. Third, we did not query the cohabitation or marital status of our study participants, and this may reflect underlying characteristics such as parity. Fourth, the data collected from 2017 correspond with a pre-COVID-19 time window. There are not studies showing changes in patterns of MCTA usage, the use of other tracking modes, or non tracking from before and during the pandemic to after the pandemic to allow for comparisons. It is possible that more recent studies exploring this type of work may differ. Lastly, the underlying reason for tracking or not was not assessed.

### Future Work

Our results suggest that adding questions surveying menstrual health history and motivations for using MCTAs can further facilitate understanding of whether differences exist among the contributors of MCTA-sourced data and the target population. Similarly, reporting of available demographic information for the user base of an app whose data were included should be encouraged in large-scale epidemiological studies using MCTA data.

Future studies examining the association between tracking preferences and health behaviors with a larger sample size provide further insights into tracking behaviors across demographics, health behaviors and conditions, and menstrual cycle characteristics with better confounding control using multivariate models.

## Appendix

Multimedia Appendix 1 Participant characteristics.

## Abbreviations

GERD: gastrointestinal reflux disease
MCTA: menstrual cycle tracking app
OM: Ovulation and Menstruation
PCOS: polycystic ovary syndrome
PTSD: posttraumatic stress disorder

## Data Availability

The data sets generated during or analyzed during this study are available from the corresponding author on reasonable request.
