# Supplementary material for: Evaluation of Menstrual Cycle Tracking Behaviors in the Ovulation and Menstruation Health Pilot Study: Cross-Sectional Study
Source: J Med Internet Res. 2023 Oct 27;25:e42164. doi: 10.2196/42164 (PMC10638629; doi:10.2196/42164)
Supplement: Multimedia Appendix 1 [file jmir_v25i1e42164_app1.docx]

**Multimedia Appendix 1.** Participant characteristics.

**Table S1.** Tracking status by recruitment location.

| **Tracking category** | ***Clinic*** | ***Community Fair*** | ***Internet*** | ***P-value*** |
| --- | --- | --- | --- | --- |
| ***N = 263*** | *n=36* | *n=61* | *n=166* |  |
| **Non-Tracker** | 16 (44.4%) | 30 (49.2%) | 51 (30.7%) | .081 |
| **App Tracker** | 14 (38.9%) | 20 (32.8%) | 69 (41.6%) |  |
| **Other Tracker** | 6 (16.7%) | 11 (18.0%) | 46 (27.7%) |  |

**Table S2.** Health behaviors and health conditions between app users and other trackers (Comparison 2).

|  | *App user n=103* | *Other tracker n=63* | *P-value** |
| --- | --- | --- | --- |
| Smoked at least 100 cigarettes over lifetime, n (%) | 6 (6%) | 11 (17.5%) | .02 |
| Body Mass Index (BMI), n (%) |  |  | .53 |
| Underweight (<18.5 kg/m2) | 3 (2.9%) | 1 (1.6%) |  |
| Normal weight (18.5-24.9 kg/m2) | 58 (56.3%) | 39 (61.9%) |  |
| Overweight (25.0-29.9 kg/m2) | 12 (11.7%) | 11 (17.5%) |  |
| Obese (≥30.0 kg/m2) | 29 (28.2%) | 12 (19.0%) |  |
| Unknown | 1 (1.0%) | 0 (0.0%) |  |
| PCOS Diagnosis by Doctor, n (%) | 17 (17%) | 10 (15.9%) | 1.00 |
| PCOS Self-diagnosis, n (%) | 10 (9.7%) | 5 (7.9%) | .78 |
| Ever Pregnant, n (%) | 16 (15.5%) | 12 (19.0%) | .53 |
| Hormonal Contraceptives Ever Use, n (%) | 74 (71.8%) | 56 (88.9%) | .01 |
| Participant’s Rating of Current Health, n (%) |  |  | 1.00 |
| Excellent/Very Good/Good | 91 (88.3%) | 57 (90.5%) |  |
| Fair/Poor | 11 (10.7%) | 6 (9.5%) |  |
| Unknown | 1 (1.0%) | 0 (0%) |  |
| Uterine fibroids diagnosis | 6 (6%) | 5 (7.9%) | .75 |
| Endometriosis diagnosis | 1 (1%) | 3 (4.8%) | .15 |
| Premature ovarian failure diagnosis | 0 (0%) | 2 (3.2%) | .19 |
| Heartburn or GERD diagnosis | 25 (24.3%) | 14 (22.2%) | .85 |
| Eating disorder diagnosis | 13 (13%) | 13 (20.6%) | .19 |
| Hypertension diagnosis | 5 (4.9%) | 2 (3.2%) | .71 |
| High cholesterol diagnosis | 9 (8.7%) | 13 (20.6%) | .04 |
| Diabetes diagnosis | 2 (1.9%) | 3 (4.8%) | .37 |
| Non-alcoholic fatty liver disease diagnosis | 1 (1.0%) | 2 (3.2%) | .69 |
| Thyroid disease diagnosis | 4 (3.9%) | 5 (7.9%) | .34 |
| Excess prolactin diagnosis | 1 (1%) | 1 (1.6%) | .32 |
| Sleep apnea diagnosis | 5 (5%) | 1 (1.6%) | .41 |
| PTSD diagnosis | 10 (9.4%) | 10 (15.9%) | .32 |
| Chronic fatigue syndrome diagnosis | 1 (1%) | 3 (4.8%) | .16 |
| Seizure disorder diagnosis | 2 (2%) | 1 (1.6%) | 1.00 |
| Depression diagnosis | 31 (30.1%) | 24 (38.1%) | .31 |
| Anxiety or panic disorder diagnosis | 36 (35.0%) | 28 (44.4%) | .25 |

*P-values calculated using Fisher’s exact test due to small cells when prevalence was below 10%

**Table S3.** Health behaviors and health conditions between any tracker and non-trackers (Comparison 3).

|  | *Any tracker n=166* | *Non-tracker n=97* | *P-value** |
| --- | --- | --- | --- |
| Smoked at least 100 cigarettes over lifetime, n (%) | 17 (10.1%) | 15 (17%) | .24 |
| Body Mass Index (BMI), n (%) |  |  | .62 |
| Underweight (<18.5 kg/m2) | 4 (2.4%) | 3 (3.1%) |  |
| Normal weight (18.5-24.9 kg/m2) | 97 (58.4%) | 52 (53.6%) |  |
| Overweight (25.0-29.9 kg/m2) | 23 (13.9%) | 18 (18.6%) |  |
| Obese (≥30.0 kg/m2) | 41 (24.7%) | 22 (22.7%) |  |
| Unknown | 1 (0.6%) | 2 (2.1%) |  |
| PCOS Diagnosis by Doctor, n (%) | 27 (16.0%) | 10 (11%) | .20 |
| PCOS Self-diagnosis, n (%) | 15 (8.9%) | 7 (8%) | .65 |
| Ever Pregnant, n (%) | 28 (16.9%) | 14 (14.4%) | .60 |
| Hormonal Contraceptives Ever Use, n (%) | 130 (78.3%) | 87 (89.7%) | .02 |
| Participant’s Rating of Current Health, n (%) |  |  | 1.00 |
| Excellent/Very Good/Good | 148 (89.2%) | 86 (88.7%) |  |
| Fair/Poor | 17 (10.2%) | 9 (9.3%) |  |
| Unknown | 1 (0.6%) | 2 (2.1%) |  |
| Uterine fibroids diagnosis | 11 (6.5%) | 5 (5.2%) | .79 |
| Endometriosis diagnosis | 4 (2.4%) | 5 (5.2%) | .30 |
| Premature ovarian failure diagnosis | 2 (1.2%) | 0 (0.0%) | .36 |
| Heartburn or GERD diagnosis | 39 (23.5%) | 12 (12.4%) | .03 |
| Eating disorder diagnosis | 26 (15.4%) | 9 (9.3%) | .19 |
| Hypertension diagnosis | 7 (4.2%) | 2 (2.1%) | .49 |
| High cholesterol diagnosis | 22 (13.3%) | 6 (6.2%) | .10 |
| Diabetes diagnosis | 5 (3.0%) | 0 (0.0%) | .16 |
| Non-alcoholic fatty liver disease diagnosis | 3 (1.8%) | 0 (0.0%) | .41 |
| Thyroid disease diagnosis | 9 (5.4%) | 3 (3.1%) | .52 |
| Excess prolactin diagnosis | 2 (1.2%) | 0 (0.0%) | .29 |
| Sleep apnea diagnosis | 6 (3.6%) | 1 (1.0%) | .43 |
| PTSD diagnosis | 20 (11.8%) | 8 (8.2%) | .41 |
| Chronic fatigue syndrome diagnosis | 4 (2.4%) | 0 (0.0%) | .30 |
| Seizure disorder diagnosis | 3 (1.8%) | 0 (0.0%) | .30 |
| Depression diagnosis | 55 (33.1%) | 26 (26.8%) | .33 |
| Anxiety or panic disorder diagnosis | 64 (38.6%) | 25 (25.8%) | .04 |

*P-values calculated using Fisher’s exact test due to small cells when prevalence was below 10%

**Table S4.** Information regarding cycle regularity, age at menarche, and cycle frequency for the overall cohort.

|  | ***Overall Cohort*** | ***App user*** | ***Non-tracker*** | ***Other tracker*** |
| --- | --- | --- | --- | --- |
|  | *N=263* | *n=103* | *n=97* | *n=63* |
| **Age at Menarche** |  |  |  |  |
| *<=7* | 1 (0.4%) | 1 (1.0%) | 0 (0.0%) | 0 (0.0%) |
| *8-10* | 26 (9.9%) | 12 (11.7%) | 8 (8.2%) | 6 (9.5%) |
| *11* | 53 (20.2%) | 19 (18.4%) | 22 (22.7%) | 12 (19.0%) |
| *12* | 73 (27.8%) | 31 (30.1%) | 24 (24.7%) | 18 (28.6%) |
| *13* | 52 (19.8%) | 24 (23.3%) | 17 (17.5%) | 11 (17.5%) |
| *14* | 25 (9.5%) | 7 (6.8%) | 11 (11.3%) | 7 (11.1%) |
| *15* | 15 (5.7%) | 6 (5.8%) | 6 (6.2%) | 3 (4.8%) |
| *16+* | 11 (4.2%) | 2 (1.9%) | 5 (5.2%) | 4 (6.3%) |
| *Missing* | 7 (2.7%) | 1 (1.0%) | 4 (4.1%) | 2 (3.2%) |
| **Time to cycle regularity** |  |  |  |  |
| *<1 year* | 134 (51.0%) | 59 (57.3%) | 45 (46.4%) | 30 (47.6%) |
| *1-2 years* | 49 (18.6%) | 17 (16.5%) | 20 (20.6%) | 12 (19.0%) |
| *3-4 years* | 14 (5.3%) | 5 (4.9%) | 5 (5.2%) | 4 (6.3%) |
| *5+* | 14 (5.3%) | 6 (5.8%) | 5 (5.2%) | 3 (4.8%) |
| *Never* | 48 (18.3%) | 15 (14.6%) | 19 (19.6%) | 14 (22.2%) |
| *Missing* | 4 (1.5%) | 1 (1.0%) | 3 (3.1%) | 0 (0.0%) |
| **Period regularity** |  |  |  |  |
| *<24 days* | 17 (6.5%) | 5 (4.9%) | 4 (4.1%) | 8 (12.7%) |
| *24-38 days* | 181 (68.8%) | 74 (71.8%) | 60 (61.9%) | 47 (74.6%) |
| *>=38* | 19 (7.2%) | 11 (10.7%) | 4 (4.1%) | 4 (6.3%) |
| *Missing* | 46 (17.5%) | 13 (12.6%) | 29 (29.9%) | 4 (6.3%) |
| **Has there ever been a time when your menstrual period was NOT regular or predictable** |  |  |  |  |
| *No* | 103 (39.2%) | 44 (42.7%) | 31 (32.0%) | 28 (44.4%) |
| *Yes* | 158 (60.1%) | 59 (57.3%) | 64 (66.0%) | 35 (55.6%) |
| *Missing* | 2 (0.8%) | 0 (0.0%) | 2 (2.1%) | 0 (0.0%) |
| **Categorized bleed day duration** |  |  |  |  |
| *0-3 days* | 116 (44.1%) | 40 (38.8%) | 50 (51.5%) | 26 (41.3%) |
| *4-7 days* | 144 (54.8%) | 62 (60.2%) | 45 (46.4%) | 37 (58.7%) |
| *Missing* | 3 (1.1%) | 1 (1.0%) | 2 (2.1%) | 0 (0.0%) |

**Table S5.** Information regarding cycle regularity, age at menarche, and cycle frequency comparing app-users to other-trackers (Comparison 2).

|  | ***App user*** | ***Other tracker*** | ***P-value*** |
| --- | --- | --- | --- |
|  | *n=103* | *n=63* |  |
| **Age at Menarche** |  |  | .72 |
| *<=7* | 1 (1.0%) | 0 (0.0%) |  |
| *8-10* | 12 (11.7%) | 6 (9.5%) |  |
| *11* | 19 (18.4%) | 12 (19.0%) |  |
| *12* | 31 (30.1%) | 18 (28.6%) |  |
| *13* | 24 (23.3%) | 11 (17.5%) |  |
| *14* | 7 (6.8%) | 7 (11.1%) |  |
| *15* | 6 (5.8%) | 3 (4.8%) |  |
| *16+* | 2 (1.9%) | 4 (6.3%) |  |
| *Missing* | 1 (1.0%) | 2 (3.2%) |  |
| **Time to cycle regularity** |  |  | .67 |
| *<1 year* | 59 (57.3%) | 30 (47.6%) |  |
| *1-2 years* | 17 (16.5%) | 12 (19.0%) |  |
| *3-4 years* | 5 (4.9%) | 4 (6.3%) |  |
| *5+* | 6 (5.8%) | 3 (4.8%) |  |
| *Never* | 15 (14.6%) | 14 (22.2%) |  |
| *Missing* | 1 (1.0%) | 0 (0.0%) |  |
| **Period regularity** |  |  | .16 |
| *<24 days* | 5 (4.9%) | 8 (12.7%) |  |
| *24-38 days* | 74 (71.8%) | 47 (74.6%) |  |
| *>=38* | 11 (10.7%) | 4 (6.3%) |  |
| *Missing* | 13 (12.6%) | 4 (6.3%) |  |
| **Has there ever been a time when your menstrual period was NOT regular or predictable** |  |  | .83 |
| *No* | 44 (42.7%) | 28 (44.4%) |  |
| *Yes* | 59 (57.3%) | 35 (55.6%) |  |
| *Missing* | 0 (0.0%) | 0 (0.0%) |  |
| **Categorized bleed day duration** |  |  | .87 |
| *0-3 days* | 40 (38.8%) | 26 (41.3%) |  |
| *4-7 days* | 62 (60.2%) | 37 (58.7%) |  |
| *Missing* | 1 (1.0%) | 0 (0.0%) |  |

*P-values calculated using Fisher’s exact test due to small cells when prevalence was below 10%

**Table S6.** Information regarding cycle regularity, age at menarche, and cycle frequency comparing any trackers to non-trackers (Comparison 3).

|  | ***Any tracker*** | ***Non-tracker*** | ***P-value*** |
| --- | --- | --- | --- |
|  | *n=166* | *n=97* |  |
| **Age at Menarche** |  |  | .85 |
| *<=7* | 1 (0.6%) | 0 (0.0%) |  |
| *8-10* | 18 (10.8%) | 8 (8.2%) |  |
| *11* | 31 (18.7%) | 22 (22.7%) |  |
| *12* | 49 (29.5%) | 24 (24.7%) |  |
| *13* | 35 (21.1%) | 17 (17.5%) |  |
| *14* | 14 (8.4%) | 11 (11.3%) |  |
| *15* | 9 (5.4%) | 6 (6.2%) |  |
| *16+* | 6 (3.6%) | 5 (5.2%) |  |
| *Missing* | 3 (1.8%) | 4 (4.1%) |  |
| Time to cycle regularity |  |  | .90 |
| *<1 year* | 89 (53.6%) | 45 (46.4%) |  |
| *1-2 years* | 29 (17.5%) | 20 (20.6%) |  |
| *3-4 years* | 9 (5.4%) | 5 (5.2%) |  |
| *5+* | 9 (5.4%) | 5 (5.2%) |  |
| *Never* | 29 (17.5%) | 19 (19.6%) |  |
| *Missing* | 1 (0.6%) | 3 (3.1%) |  |
| Period regularity |  |  | .43 |
| *<24 days* | 13 (7.8%) | 4 (4.1%) |  |
| *24-38 days* | 121 (72.9%) | 60 (61.9%) |  |
| *>=38* | 15 (9.0%) | 4 (4.1%) |  |
| *Missing* | 17 (10.2%) | 29 (29.9%) |  |
| Has there ever been a time when your menstrual period was NOT regular or predictable |  |  | .09 |
| *No* | 72 (43.4%) | 31 (32.0%) |  |
| *Yes* | 94 (56.6%) | 64 (66.0%) |  |
| *Missing* | 0 (0.0%) | 2 (2.1%) |  |
| Categorized bleed day duration |  |  | .05 |
| *0-3 days* | 66 (39.8%) | 50 (51.5%) |  |
| *4-7 days* | 99 (59.6%) | 45 (46.4%) |  |
| *Missing* | 1 (0.6%) | 2 (2.1%) |  |

*P-values calculated using Fisher’s exact test due to small cells when prevalence was below 10%

**Table S7.** Information regarding hormonal contraceptive use between app-users and non-trackers amongst those that have ever used hormonal contraceptives (Comparison 2).

|  | ***App user*** | ***Other tracker*** | ***P-value*** |
| --- | --- | --- | --- |
| *Ever used hormonal contraceptives* | *n=74* | *n=56* |  |
| Current use of hormonal contraceptives | 31 (42%) | 37 (66%) | .006 |
| Use of hormonal contraceptives to regulate period | 25 (34%) | 30 (54%) | .02 |

**Table S8.** Information regarding hormonal contraceptive use between app-users and non-trackers amongst those that have ever used hormonal contraceptives (Comparison 3).

|  | ***Any tracker*** | ***Non-tracker*** | ***P-value*** |
| --- | --- | --- | --- |
| *Ever used hormonal contraceptives* | *n=74* | *n=87* |  |
| Current use of hormonal contraceptives | 68 (52%) | 63 (72%) | .003 |
| Use of hormonal contraceptives to regulate period | 55 (42%) | 41 (47%) | .48 |

**Table S9.** Information regarding hormonal contraceptive use for the overall cohort amongst those that have ever used hormonal contraceptives.

|  | ***Overall Cohort*** | ***App user*** | ***Non-tracker*** | ***Other tracker*** |
| --- | --- | --- | --- | --- |
| *Ever used hormonal contraceptives* | *N=217* | *n=74* | *n=87* | *n=56* |
| Current use of hormonal contraceptives | 131 (60%) | 31 (42%) | 63 (72%) | 37 (66%) |
| Use of hormonal contraceptives to regulate period | 96 (44%) | 25 (34%) | 41 (47%) | 30 (54%) |
